# Associations of Lifestyle, Medication, and Socio-Demographic Factors with Disability in People with Multiple Sclerosis: An International Cross-Sectional Study

**DOI:** 10.1371/journal.pone.0161701

**Published:** 2016-08-25

**Authors:** George A. Jelinek, Alysha M. De Livera, Claudia H. Marck, Chelsea R. Brown, Sandra L. Neate, Keryn L. Taylor, Tracey J. Weiland

**Affiliations:** Neuroepidemiology Unit, Melbourne School of Population and Global Health, The University of Melbourne, Melbourne, VIC, Australia; Medizinische Universitat Innsbruck, AUSTRIA

## Abstract

**Objective:**

Emerging evidence links modifiable lifestyle risk factors to disease progression in multiple sclerosis (MS). We sought further evidence around this hypothesis through detailed analysis of the association with disability of lifestyle behaviours of a large international sample of people with MS.

**Materials and Methods:**

A total of 2469 people with MS from 57 countries provided self-reported data via cross-sectional online survey on lifestyle (mostly with validated tools) and the primary outcome measure, disability (Patient Determined Disease Steps), categorised from 8 steps into 3 categories, mild, moderate and major disability. Multinomial logistic regression modelling derived relative risk ratios (RRRs) for disability categories.

**Results:**

RRRs of having moderate vs mild disability were: diet (per 30 points on 100 point scale) 0.72 (95%CI 0.52–0.98), ever smoking 1.32 (1.06–1.65), exercise (moderate/high vs low) 0.35 (0.28–0.44), latitude (per degree from the equator) 1.02 (1.01–1.04), and number of comorbidities (2 vs none) 1.43 (1.04–1.95), (3 vs none) 1.56 (1.13–2.16). RRRs of having major vs mild disability were: exercise (moderate/high vs low) 0.07 (0.04–0.11), alcohol consumption (moderate vs low) 0.45 (0.30–0.68), plant-based omega 3 supplementation 0.39 (0.18–0.86), and disease-modifying medication use 0.45 (0.29–0.70).

**Conclusions:**

Healthier lifestyle has strong associations with disability in our large international sample of people with MS, supporting further investigation into the role of lifestyle risk factors in MS disease progression.

## Introduction

Multiple sclerosis (MS) is an incurable, lifelong disease of the central nervous system that is usually progressively disabling. Genetic background is a key risk factor for the development of MS, accounting for around a quarter of the risk.[1] However, genome-wide associations studies to date have found no genetic associations with disease course or progression of disability.[2] Thus, environmental factors, many of them potentially modifiable, might play a central role in disease progression, and could form the basis of a secondary preventive approach to disease management.

A rapidly expanding literature, reviewed elsewhere,[3] has hypothesised that lifestyle factors are key determinants of MS disease progression. Compelling hypotheses are emerging linking MS pathogenesis and progression to lipid dysregulation[4] and developed-world lifestyles, particularly nutrition,[5] sedentary behaviour,[6] and stress.[7–8] While a range of immunomodulatory disease-modifying medications that reduce rate of relapses by around 30–50% has become available over the last two decades, evidence supporting a significant impact on disease progression is still not strong.[9] Relapse rates have been considered primary endpoints in many short-term industry-funded studies, however disability is clearly a more important outcome.[10]

Our central hypothesis has been that a secondary preventive approach to disease progression and accumulation of disability is feasible, based on attention to modifiable lifestyle risk factors.[3, 11, 12] While considerable available evidence supports this contention, to date, there have been few major population studies of people with MS that have included examination of a range of lifestyle risk factors and disability status, allowing analysis of the relative strengths of these associations. We sought therefore to collect detailed lifestyle data from a large international sample of people with MS and undertake regression modelling to assess their association with disability status. A secondary outcome was to assess these associations with self-reported physician-diagnosed relapse rate in the year leading up to the survey.

While understanding and acknowledging important issues of temporality when assessing current disability, historical relapse-rate and recent lifestyle behaviours, and that cross-sectional analysis precludes determination of causation, we sought to gather evidence to test our hypothesis. This would potentially allow determination of the relative strength of association between each of the lifestyle risk factors and disability, informing further epidemiological and clinical trial research, and provide important information for people with MS and clinicians seeking to offer lifestyle advice for their patients.

## Materials and Methods

### Participants and data collection

We have previously described the Health Outcomes and Lifestyle Interventions in a Sample of people with Multiple sclerosis (HOLISM) study in detail.[11] In short, we approached potential participants with definite or possible MS via Web 2.0 platforms, including MS society online forums and websites, and social media groups such as Facebook, to respond to a cross-sectional survey via Survey Monkey®.

All data were self-reported. We asked for information on demographics including age, gender, marital status, educational status, and occupational status, with researcher-devised questions. Reported height and weight were used to calculate body mass index (BMI) according to World Health Organisation (WHO) criteria (<20 underweight, 20-<25 normal weight, 25-<30 overweight and ≥30 obese). Co-morbidities were assessed with The Self-Administered Comorbidity Questionnaire (SCQ).[13] We slightly modified the Dietary Habits Questionnaire (DHQ),[14] developed for diet measurement in a cardiac rehabilitation population, to assess diet on a 0–100 scale. Social support was measured with the Single Item Measure of Social Support.[15]

We used the International Physical Activity Questionnaire (IPAQ) to measure the level of exercise participants were undertaking.[16] IPAQ levels briefly were described as: high active (vigorous-intensity activity on at least 3 days, or 7 days of walking, or moderate or vigorous activity); moderately active (3 or more days of vigorous activity of at least 20 minutes per day, or 5 or more days of moderate or higher intensity activity or walking of at least 30 minutes per day); or low active (less active than the moderate category). Due to lack of available validated measures, we used researcher-devised items to measure omega-3 supplementation (plant- or fish-based oils or both), vitamin D supplementation (low: average <5000IU/day, high: average ≥5000IU/day), frequency and amount of meditation practice, alcohol consumption (low: <15g/week], moderate: up to 30g/day for women and 45g/day for men, or high) and current and former smoking status.

#### Exclusion criteria

A total of 3132 people with definite or possible MS commenced the survey. Of these, we excluded 663 who did not have confirmed MS; this left 2469 respondents for analysis (whole sample) and a sub-sample of 1493 respondents with relapsing-remitting MS type (relapsing-remitting sample). Participants were English-speaking and 18 years or older.

### Measuring disability and relapses

To measure disability, we used the well-validated Patient Determined Disease Steps (PDDS) which is scored ordinally from 0 (normal) to 8 (bed bound), is easy to understand and administer, and correlates well with the EDSS.[17] The tool has strong concordance between raters and has been considered a practical tool to assess disability over time,[18] including its use in the large North American Research Committee On Multiple Sclerosis (NARCOMS) Study.[19] In order to enhance clinical interpretability, the eight steps of PDDS were grouped into three categories: steps 0–2 representing no limitations to walking, but potentially other limitations, were classified as low disability; steps 3–5 representing limitations to walking up to ability to walk 25 feet in 20 seconds without a cane or crutch, but cane or crutch for anything further, classified as moderate disability; and steps 6–8 representing bilateral cane or crutch support, wheelchair or bedbound as major disability. In addition, we asked whether participants had experienced any physician-diagnosed relapses over the 12 months leading up to the survey.

### Ethics, consent and permissions

Respondents were first taken to a participant information and consent page, and those providing consent were directed to a detailed online questionnaire incorporating validated tools where possible. The Health Sciences Human Ethics Sub-Committee at the University of Melbourne provided ethical approval for the study (Ethics ID: 1545102).

### Statistical analysis

Data were analysed using Stata, V12 (StataCorp, College Station, Texas, USA) and R (R Foundation for Statistical Computing, Vienna, Austria). Continuous data are presented as mean and standard deviation, and categorical data are presented as percentages with frequency. Skewed data are presented as median with interquartile range (25th-75th centile) and log transformed for regression analyses. We used multivariable logistic and multinomial logistic regression models to explore associations of a range of lifestyle factors with disability and any relapses respectively. In doing so, we adjusted for confounders (e.g., age and gender) identified *a priori* using our subject-matter knowledge from previous studies.[20–24] We tested for potential interaction terms using likelihood ratio tests, and found no evidence of interactions. For the fitted models, we assessed whether the probabilities estimated by the models agree with the observed outcomes using Hosmer–Lemeshow test.[25] Associations from multinomial logistic regression models are presented as odds as indicated for relative risk ratios (RRRs).

We report descriptive statistics (Table 1) and both complete case analysis (analysis restricted to respondents with available data: see Tables 2 and 3) and analysis using multiple imputation (S1 and S2 Tables). For multiple imputation, Fully Conditional Specification[26] with a single imputation model consisting of the variables in the analysis model as well as auxiliary variables (variables not included in the analysis model, but either correlated with the missing variables or associated with missingness of the variables) was used.[27] Fifty multiply imputed datasets were created, and results from these 50 datasets combined using Rubin’s rules.[27]

**Table 1:** Characteristics of outcome variables and the lifestyle factors.

	Sample Characteristics	
Factor	Values	
	Relapsing-remitting (N = 1493)	Whole sample (N = 2469)
Disability^a^		
Low	983/1413 (69.6)	1267/2300 (55.1)
Moderate	388/1413 (27.5)	793/2300 (34.5)
Major	42/1413 (2.9)	240/2300 (10.4)
Years since diagnosis^c^	5 (3,10)	6 (3,12)
Any relapse^a^		
Yes	672/1452 (46.3)	939/2298 (40.9)
DMD use^a^		
taken at least 12 months	591/1365 (43.3)	759/2232 (34.0)
Latitude (degrees) ^b^	40.9 (8.8)	41.0 (8.7)
DHQ^b^	79.2 (12.3)	78.9 (12.1)
Smoker^a^		
current or former	697/1400 (49.8)	1191/2290 (52.0)
Alcohol^a^		
Low	838/1391 (60.2)	1398/2275 (61.5)
moderate or high	553/1391 (39.8)	877/2275 (38.5)
Vitamin D supplementation		
Low	1075/1345 (79.9)	1747/2194 (79.6)
High	270/1345 (20.1)	447/2194 (20.4)
IPAQ^a^		
Low	462/1362 (33.9)	922/2232 (41.3)
moderate or high	900/1362 (66.1)	707/2232 (58.7)
Omega 3 supplementation		
None	494/1350 (36.6)	806/2199 (36.6)
flaxseed only	129/1350 (9.6)	204/2199 (9.3)
Other	727/1350 (53.8)	2199/2199 (54.1)
Meditation^a^		
Never	971/1368 (71.0)	1568/2243 (69.9)
once a week or more	397/1368 (29.0)	675/2243 (30.1)
BMI^a^		
Normal	780/1478 (52.8)	1302/2425 (53.7)
Underweight	60/1478 (4.1)	104/2425 (4.3)
Overweight	323/1478 (21.8)	548/2425 (22.6)
Obese	315/1478 (21.3)	471/2425 (19.4)
Age^b^ (years)	42.7 (9.6)	45.5 (10.5)
Gender^a^		
Male	219/1412 (15.5)	407/2303 (17.7)
Employment^a^		
Unemployed	108/1453 (7.4)	196/2405 (8.2)
student or stay at home carer	169/1453 (11.6)	244/2405 (10.2)
employed full or part time	930/1453 (64.0)	1323/2405 (55.0)
retired due to age or disability	246/1453 (16.9)	642/2405 (26.7)
Marital status^a^		
Single	239/1481 (16.1)	359/2431 (14.8)
Married	1106/1481 (74.7)	1788/2431 (73.6)
Separated	136/1481 (9.2)	284/2431 (11.7)

^a^Values are number (%)

^b^Values are mean (SD)

^c^Values are median (25th–75th percentile), Denominators vary reflecting number of respondents completing each item

**Table 2:** Associations between lifestyle factors and disability^#^.

	Moderate disability			Major disability		
	RRR	95% CI	p-value	RRR	95% CI	p-value
Latitude (degrees)	**1.02**	**(1.01,1.04)**	**<0.001**	1.02	(1.00,1.04)	0.08
BMI	Reference					
Underweight	1.04	(0.58,1.84)	0.9	0.63	(0.24,1.63)	0.34
Overweight	1	(0.76,1.32)	0.99	0.75	(0.46,1.22)	0.25
Obese	1.06	(0.78,1.44)	0.73	1.08	(0.66,1.79)	0.75
Alcohol consumption						
Moderate or high	0.83	(0.66,1.04)	0.11	**0.45**	**(0.30,0.68)**	**<0.001**
Comorbidities						
None	Reference					
One	1.21	(0.9,1.63)	0.2	0.93	(0.56,1.54)	0.78
Two	**1.43**	**(1.04,1.95)**	**0.03**	0.84	(0.49,1.43)	0.52
Three or more	**1.56**	**(1.13,2.16)**	**0.01**	0.75	(0.43,1.31)	0.30
DHQ (per 30 points)	**0.72**	**(0.52,0.98)**	**0.04**	0.63	(0.37,1.07)	0.09
Smoker						
No	Reference					
Current or former	**1.32**	**(1.06,1.65)**	**0.01**	1.03	(0.70,1.50)	0.89
Vitamin D supplementation						
low	Reference					
high	1.17	(0.88,1.55)	0.29	1.06	(0.75,1.48)	0.76
IPAQ						
low	Reference					
mod/high	**0.35**	**(0.28,0.44)**	**<0.001**	**0.07**	**(0.04,0.11)**	**<0.001**
Omega3 supplementation						
none	Reference					
flaxseed only	0.95	(0.63,1.44)	0.81	**0.39**	**(0.18,0.86)**	**0.02**
other	0.77	(0.60,1.00)	0.05	**0.63**	**(0.41,0.96)**	**0.03**
DMD use,						
No	Reference					
Yes (>12 months)	0.79	(0.62,1.00)	0.05	**0.45**	**(0.29,0.70)**	**<0.001**

^#^ Relative risk ratios (RRR) and 95% Confidence Intervals (CI) obtained using multivariable logistic regression, model adjusted for age, gender, and years since diagnosis. Statistically significant associations at a significance level of 0.05 are shown in bold.

**Table 3:** Associations between lifestyle factors and any relapses^#^.

Factor	OR	95% CI	p-value
Disability			
Low	Reference		
Moderate	**1.8**	**(1.31,2.47)**	**<0.001**
Major	1.95	(0.86,4.38)	0.11
Latitude (degrees)	1.00	(0.99,1.02)	0.83
BMI	0.62	(0.31,1.20)	0.17
Underweight			
Overweight	1.17	(0.85,1.62)	0.33
Obese	1.09	(0.77,1.56)	0.62
Alcohol consumption			
Moderate or high	1.00	(0.77,1.30)	0.99
Comorbidities			
None	Reference		
One	1.16	(0.83,1.61)	0.38
Two	**1.68**	**(1.16,2.44)**	**0.01**
Three or more	**2.38**	**(1.63,3.49)**	**<0.001**
DHQ (per 30 points)	**0.67**	**(0.46,0.97)**	**0.03**
Smoker			
No	Reference		
Current or former	0.9	(0.7,1.17)	0.43
Vitamin D supplementation			
low	Reference		
high	0.82	(0.59,1.13)	0.23
IPAQ			
low	Reference		
high	0.98	(0.74,1.30)	0.88
Omega3 supplementation			
none	Reference		
flaxseed only	**0.56**	**(0.33,0.92)**	**0.02**
other	1.3	(0.97,1.74)	0.1
DMD use			
Not taken >12 months	Reference		
Taken >12 months	**0.45**	**(0.35,0.59)**	**<0.001**

^#^ Odds ratios (OR) and 95% Confidence Intervals (CI) obtained using multivariable logistic regression, model adjusted for age, gender and years since diagnosis. Statistically significant associations at a significance level of 0.05 are shown in bold.

## Results

### Sample characteristics

Our whole sample represented people with MS from 57 countries, the majority from North America, with significant proportions from Australasia and Europe (Table 1). Women were over-represented in a ratio of over 4:1. Participants were well educated and had unusually healthy lifestyles. Major disability, at around 10% of the sample, was lower than in comparable groups previously studied.[28] Their diet was very healthy, scoring on average 79 of a possible 100 points on the DHQ, with only 11.7% current smokers and very few heavy alcohol drinkers (19 in the overall sample), and nearly 60% of the sample was moderately or highly physically active. Around a third meditated regularly and the majority supplemented with omega 3 fatty acids and vitamin D. There were some differences in demographics, disease characteristics and lifestyle behaviours between the whole sample and those in the relapsing-remitting group as expected, particularly in relation to degree of disability (Table 1).

### Disability

Stable and modifiable factors with significant associations with disability and relapse rate in univariate analyses were included in regression models.

Stable and relatively stable factors with significant associations with disability were gender, age, years since diagnosis, latitude and number of comorbidities (Table 2). For each additional log year since diagnosis, the odds of being in the moderate versus low disability group increased by a factor of 1.66 (95% CI: 1.44, 1.91) and being in the major versus low disability group increased by a factor of 3.68 (95% CI: 2.80, 4.83) given the other variables were held constant. For those participants with two co-morbidities vs none and three or more co-morbidities vs none, the odds for moderate relative to low disability increased by 43% and 56% respectively. For every 10 degrees of latitude further away from the equator, the odds for being in the moderate versus low disability group increased by 20%.

Of the modifiable lifestyle factors, better diet, non–smoking, more exercise, omega 3 supplementation and disease-modifying drug use were associated with lower disability. For diet, every 30 point improvement on the 100 point DHQ was associated with 28% lower odds of being in the moderate compared to low disability group. Being a current or a former smoker was associated with 32% higher odds of being in the moderate compared to low disability group. Moderate/high versus low alcohol consumption was associated with 55% lower odds of moderate compared to low disability; we included high consumption with moderate due to the very low numbers in the high group precluding meaningful statistical analysis. Increasing amounts of physical activity were associated with 65% and 93% lower odds of being in the moderate and major disability groups respectively compared to low disability group.

Those participants taking plant-based omega 3 supplements had 61% lower odds of being in the major compared to low disability group. However, as we have previously seen in analyses not adjusted for all other lifestyle factors,[21] strong associations between those taking fish-based omega 3 supplements, or both plant- and fish-based oils were not found (see multiple imputation results in S1 Table). Participants taking a disease-modifying drug for longer than 12 months were 55% less likely to be in the major versus low disability category.

### Relapses

In the group with relapsing-remitting MS, we found fewer associations of lifestyle factors with any relapses in the last year (Table 3). The odds of having any relapses versus not were reduced by a factor of 0.58 (95% CI: 0.49, 0.69) with each log year since diagnosis. Having moderate compared to low disability was associated with 80% increased odds of relapse. Having two, or three or more comorbidities was associated with 68% and 138% increased odds of relapses respectively. For every 30 points improvement in diet on the DHQ, there were 33% lower odds of having had any relapse in the previous 12 months, although this association became marginally non-significant on multiple imputation (S2 Table). Taking plant-based omega 3 supplements was associated with 44% lower odds of relapse, and taking a disease-modifying medication over the previous 12 months with 55% lower odds.

## Discussion

Notwithstanding important limitations in temporality, our data provide, for the first time, an opportunity to examine how potentially modifiable lifestyle and relatively stable socio-demographic risk factors are associated with disability in a large sample of people with all types of MS from all over the world. To our knowledge, no group has yet examined a full suite of lifestyle factors in the same model as known socio-demographic determinants of disability with mutual adjustment for these factors. In keeping with our hypothesis, our data provide evidence of the potential influence of these lifestyle factors on disability in MS.

Predictably, we found more disability in older people with a longer duration of disease; we also found that these people were less likely to have suffered relapses over the previous year. Our data show that women were less likely to be disabled, as has previously been suggested.[29] Our fully adjusted data showed increased disability with increasing distance from the equator, in keeping with our previous work.[30] Increasing number of co-morbidities, potentially modifiable with healthy lifestyle, showed a dose-response effect with greater likelihood of disability and relapses over the past year, in line with prospective Australian data.[31]

The importance of our study however, lies in the strengthening of the evidence base supporting the potential role of lifestyle risk factors in disability accumulation, while controlling for other factors including disease-modifying drug use. Despite the passage of many decades since Swank’s seminal work in an intervention study of low saturated fat diet in a cohort of people with MS followed closely for 34 years,[32] little real progress has been made in clearly elucidating the role of diet in disease progression. Our data show significant independent associations of better diet with lower disability, in a dose-response fashion, and a similar size marginal association with likelihood of having had a relapse in the year prior to the study. For those eating a poor diet, as many in developed countries do, a 30 point improvement in diet score on the DHQ is entirely feasible. Such a difference is associated with 28% lower likelihood of being moderately disabled and marginally significant 33% lower risk of relapse over the prior year in our analysis. While more epidemiological and intervention studies are clearly needed to add to this evidence and for dietary change to become part of a secondary preventive strategy, given the other health benefits that accrue from dietary improvement, including potential reduction in number of co-morbidities, many clinicians may be inclined to offer such advice and support to their patients.

Our data are in line with a recent study showing benefits of smoking cessation in preventing disability for people with MS.[33] Again, smoking cessation also reduces the risk of other co-morbidities, and should now be a standard recommendation for people with MS as part of a comprehensive secondary prevention program. Our findings on alcohol are congruent with other medical literature on possible beneficial effects of moderate alcohol consumption,[34, 35] although this has been questioned and many potential confounders may explain the association.[36] At the least, our data suggest that people with MS can be reasonably reassured that moderate alcohol consumption is unlikely to be harmful. We had very few heavy drinkers in our sample, so it was not statistically sensible to analyse them separately, although our previous work suggests they have poor outcomes.[24, 37]

While there is a significant likelihood of some contribution of reverse causality in our findings on the strong associations of more exercise with less disability, this is to some extent mitigated by adjustment for years since diagnosis and age in the model. It remains very plausible, in keeping with randomised controlled trials,[38] that regular exercise may have an impact on disability in MS. In line with our previous work,[21] we have now found independent and strong associations of plant-based omega 3 supplementation with disability, and also with relapse rate, but found equivocal or no association for fish oil. Supplementation with plant-based rather than fish oil is clearly indicated in future intervention studies. While speculative, it is possible that poor commercial storage of fish oils leads to oxidation and lack of efficacy;[37] plant-based omega 3s such as flaxseed oils are routinely cold-pressed and refrigerated before sale.

Our data also provide real-world evidence about how the commonly prescribed disease-modifying drugs are performing in practice, outside of the less translatable environment of clinical trials. We found significant associations of taking disease-modifying medications over the previous 12 months with lower likelihood of relapse over that time, and current major disability. This supports other observational studies with similar findings,[39] although some have not found these associations.[40] While there may have been some contribution from reverse causation, particularly in that those more disabled would be less likely to be offered these drugs, the data may provide some reassurance for people with MS and their clinicians, that these medications are likely to be influencing not only relapses, but potentially the course of the disease.

### Strengths and limitations

Our study has a number of strengths and unique features. No other study has examined such a large range of modifiable risk factors in the one model. Due to our sample selection, biased towards those seeking information and support about potential changes they could make to affect disease progression, we had a full range of exposures for most lifestyle factors. For example around 30% consumed no meat and no dairy respectively, most exercised regularly, only around 10% smoked, and around 30% meditated. While this limits generalizability to an extent, it provides greater opportunity to detect important associations; a sample comprising largely those eating poorly, not exercising and not addressing stress would have been significantly less likely to uncover these associations.

Our study however has significant limitations, apart from this selection bias. Most importantly, the contribution of reverse causality to magnitude and presence of association cannot be quantified, but is likely for at least some of the lifestyle variables studied, such as physical activity. All data were self-reported, with the nature of recruitment prohibiting data verification. This raises the potential for the influence of several biases, including recall and measurement bias. We were not able to locate validated measures for a number of the exposures and the relapse outcome, and thus several are researcher-devised, and have not been validated elsewhere. This may have limited our potential to find associations of significance for relapse rate, as the measure was subject to biases and relatively crude.

Temporality is also a major limitation of the study; as disability often takes many years to accrue, and our study was cross-sectional, the data allow examination only of current lifestyle in relation to an outcome of potentially years’ standing. As we are currently following up this cohort at 2.5 yearly intervals, the associations we have seen in this paper can be further examined using longitudinal data.

### Conclusion

In a large international sample of people with MS, modifiable lifestyle factors, particularly diet, smoking, alcohol consumption, physical activity, and plant-based omega 3 supplementation, each have strong independent associations with disability after adjustment for socio-demographic factors and disease-modifying drug use. Diet and plant-based omega 3 supplements are also associated with fewer relapses. Disease-modifying drug use is independently associated with lower disability and fewer relapses. This evidence supports further investigation into the role of lifestyle risk factors in MS disease progression, and the trialling of a comprehensive secondary prevention program for people with MS. Relevant lifestyle risk factor modification, in combination with disease-modifying drug therapy may be the optimal management for people with MS.

## Supporting Information

S1 Table Associations between lifestyle factors and disability using multiply imputed data. Relative risk ratios (RRR) and 95% Confidence Intervals (CI) obtained using multivariable logistic regression on multiply imputed data, model adjusted for age, gender and years since diagnosis. Statistically significant associations at a significance level of 0.05 are shown in bold.
(DOCX)

S2 Table Associations between lifestyle factors and any relapses using multiply imputed data. Odds ratios (OR) and 95% Confidence Intervals (CI) obtained using multivariable logistic regression on multiply imputed data, model adjusted for age, gender, and years since diagnosis. Statistically significant associations at a significance level of 0.05 are shown in bold.
(DOCX)

## References

[1] Sadovnick AD, Ebers GC, Dyment DA, Risch NJ. Evidence for genetic basis of multiple sclerosis. The Canadian Collaborative Study Group. Lancet. 1996;347(9017):1728–30. 8656905 10.1016/s0140-6736(96)90807-7

[2] Sawcer S, Hellenthal G, Pirinen M, Spencer CC, Patsopoulos NA, Moutsianas L, et al Genetic risk and a primary role for cell-mediated immune mechanisms in multiple sclerosis. Nature. 2011;476(7359):214–9. 10.1038/nature10251 21833088 PMC3182531

[3] Jelinek G. Overcoming Multiple Sclerosis. The evidence-based 7 step recovery program. Sydney: Allen and Unwin; 2016.

[4] Corthals AP. Multiple sclerosis is not a disease of the immune system. Q Rev Biol. 2011;86(4):287–321. 22384749 10.1086/662453

[5] Riccio P, Rossano R. Nutrition facts in multiple sclerosis. ASN Neuro. 2015;7(1). 10.1177/1759091414568185 PMC4342365 25694551

[6] Motl RW, Sandroff BM. Benefits of Exercise Training in Multiple Sclerosis. Curr Neurol Neurosci Rep. 2015;15(9):62 10.1007/s11910-015-0585-6 26223831

[7] Mitsonis CI, Potagas C, Zervas I, Sfagos K. The effects of stressful life events on the course of multiple sclerosis: a review. Int J Neuroscience. 2009;119(3):315–35. 10.1080/00207450802480192 19116839

[8] Grant WB, Riise T. Multiple sclerosis: A lifestyle disease? Neurology. 2016. 10.1212/WNL.0000000000002487 26962071

[9] Wingerchuk DM, Carter JL. Multiple sclerosis: current and emerging disease-modifying therapies and treatment strategies. Mayo Clin Proc. 2014;89(2):225–40. 10.1016/j.mayocp.2013.11.002 24485135

[10] Ebers GC, Heigenhauser L, Daumer M, Lederer C, Noseworthy JH. Disability as an outcome in MS clinical trials. Neurol. 2008. 10.1212/01.wnl.0000313034.46883.16 18480462

[11] Hadgkiss EJ, Jelinek GA, Weiland TJ, Pereira NG, Marck CH, van der Meer DM. Methodology of an international study of people with multiple sclerosis recruited through web 2.0 platforms: demographics, lifestyle, and disease characteristics. Neurol Res Int. 2013:2013:580596 10.1155/2013/580596 23691313 PMC3649686

[12] Jelinek GA, Hassed CS. Managing multiple sclerosis in primary care: are we forgetting something? Qual Prim Care. 2009;17(1):55–61. 19281675

[13] Sangha O, Stucki G, Liang MH, Fossel AH, Katz JN. The Self-Administered Comorbidity Questionnaire: a new method to assess comorbidity for clinical and health services research. Arthritis Rheum. 2003;49(2):156–63. 12687505 10.1002/art.10993

[14] McKellar S, Horsley P, Chambers R, Pullen M, Vendersee P, Clarke C, et al Development of the Diet Habits Questionnaire for Use in Cardiac Rehabilitation. Aust J Prim Health. 2008;14(3):43–7.

[15] Blake R, McKay D. A single-item measure of social supports as a predictor of morbidity. J Fam Pract. 1986;22(1):82–4. 3941304

[16] Craig CL, Marshall AL, Sjostrom M, Bauman AE, Booth ML, Ainsworth BE, et al International physical activity questionnaire: 12-country reliability and validity. Med Sci Sports Exercise. 2003;35(8):1381–95. 10.1249/01.MSS.0000078924.61453.FB 12900694

[17] Hohol MJ, Orav EJ, Weiner HL. Disease steps in multiple sclerosis: a simple approach to evaluate disease progression. Neurol. 1995;45(2):251–5. 10.1212/wnl.45.2.251 7854521

[18] Hohol MJ, Orav EJ, Weiner HL. Disease steps in multiple sclerosis: a longitudinal study comparing disease steps and EDSS to evaluate disease progression. Mult Scler. 1999;5(5):349–54. 10516779 10.1177/135245859900500508

[19] Marrie RA, Cutter G, Tyry T, Hadjimichael O, Campagnolo D, Vollmer T. Changes in the ascertainment of multiple sclerosis. Neurol. 2005;65(7):1066–70. 10.1212/01.wnl.0000178891.20579.64 16217060

[20] Hadgkiss EJ, Jelinek GA, Weiland TJ, Pereira NG, Marck CH, van der Meer DM. The association of diet with quality of life, disability, and relapse rate in an international sample of people with multiple sclerosis. Nutr Neurosci. 2015;18(3):125–36. 10.1179/1476830514Y.0000000117 24628020 PMC4485697

[21] Jelinek GA, Hadgkiss EJ, Weiland TJ, Pereira NG, Marck CH, van der Meer DM. Association of fish consumption and Omega 3 supplementation with quality of life, disability and disease activity in an international cohort of people with multiple sclerosis. Int J Neuroscience. 2013;123(11):792–800. 10.3109/00207454.2013.803104 PMC3821380 23713615

[22] Marck CH, Hadgkiss E, Weiland TJ, van der Meer DM, Pereira N, Jelinek GA. Physical activity and associated levels of disability and quality of life in people with multiple sclerosis: a large international survey. BMC Neurol. 2014; 14:143 10.1186/1471-2377-14-143 25016312 PMC4110931

[23] Marck CH, Neate SL, Taylor KL, Weiland TJ, Jelinek GA. Prevalence of Comorbidities, Overweight and Obesity in an International Sample of People with Multiple Sclerosis and Associations with Modifiable Lifestyle Factors. PLoS One. 2016;11(2):e0148573 10.1371/journal.pone.0148573 26849357 PMC4743906

[24] Weiland TJ, Hadgkiss EJ, Jelinek GA, Pereira NG, Marck CH, van der Meer DM. The association of alcohol consumption and smoking with quality of life, disability and disease activity in an international sample of people with multiple sclerosis. J Neurol Sci. 2014;336(1–2):211–9. 10.1016/j.jns.2013.10.046 24290614

[25] Hosmer DWJ, Lemeshow SA. Goodness-of-fit tests for the multiple logistic regression model. Communications in Statistics. 1980;A9:1043–69.

[26] Van Buuren S, Brand JP, Groothuis-Oudshoorn CGM, Rubin DB. Fully conditional specification in multivariate imputation. J Statist Comput Simul. 2006;76(12):1049–64.

[27] Schafer JL. Analysis of incomplete multivariate data Cox DR, Isham V, Keiding N, Reid N, Tong H, editors. Florida: Chapman and Hall/CRC; 1997.

[28] Marrie RA, Cutter G, Tyry T, Vollmer T, Campagnolo D. Does multiple sclerosis-associated disability differ between races? Neurol. 2006;66(8):1235–40. 10.1212/01.wnl.0000208505.81912.82 16636241

[29] Fernandez O. Integrating the tools for an individualized prognosis in multiple sclerosis. J Neurol Sci. 2013;331(1–2):10–3. 10.1016/j.jns.2013.04.021 23692966

[30] Jelinek GA, Marck CH, Weiland TJ, Pereira N, van der Meer DM, Hadgkiss EJ. Latitude, sun exposure and vitamin D supplementation: associations with quality of life and disease outcomes in a large international cohort of people with multiple sclerosis. BMC Neurol. 2015;15:132 10.1186/s12883-015-0394-1 26243188 PMC4525738

[31] Tettey P, Siejka D, Simpson S Jr, Taylor B, Blizzard L, Ponsonby AL, et al Frequency of Comorbidities and Their Association with Clinical Disability and Relapse in Multiple Sclerosis. Neuroepidemiology. 2016;46(2):106–13. 10.1159/000442203 26784322

[32] Swank RL, Dugan BB. Effect of low saturated fat diet in early and late cases of multiple sclerosis. Lancet. 1990;336(8706):37–9. 1973220 10.1016/0140-6736(90)91533-g

[33] Ramanujam R, Hedstrom AK, Manouchehrinia A, Alfredsson L, Olsson T, Bottai M, et al Effect of Smoking Cessation on Multiple Sclerosis Prognosis. JAMA Neurol. 2015:1–7. 10.1001/jamaneurol.2015.1788 26348720

[34] Zhang C, Qin YY, Chen Q, Jiang H, Chen XZ, Xu CL, et al Alcohol intake and risk of stroke: a dose-response meta-analysis of prospective studies. Int J Cardiol. 2014;174(3):669–77. 10.1016/j.ijcard.2014.04.225 24820756

[35] Roerecke M, Rehm J. Alcohol consumption, drinking patterns, and ischemic heart disease: a narrative review of meta-analyses and a systematic review and meta-analysis of the impact of heavy drinking occasions on risk for moderate drinkers. BMC Med. 2014;12:182 10.1186/s12916-014-0182-6 25567363 PMC4203905

[36] Stockwell T, Zhao J, Panwar S, Roemer A, Naimi T, Chikritzhs T. Do "Moderate" Drinkers Have Reduced Mortality Risk? A Systematic Review and Meta-Analysis of Alcohol Consumption and All-Cause Mortality. J Stud Alcohol Drugs. 2016;77(2):185–98. 26997174 10.15288/jsad.2016.77.185 PMC4803651

[37] Albert BB, Cameron-Smith D, Hofman PL, Cutfield WS. Oxidation of marine omega-3 supplements and human health. Biomed Res Int. 2013;2013:464921 10.1155/2013/464921 23738326 PMC3657456

[38] Rietberg MB, Brooks D, Uitdehaag MJB, Kwakkel G. Exercise therapy for multiple sclerosis. Cochrane database of systematic reviews. 2011(1). 10.1002/14651858.CD003980.pub2 PMC6485797 15674920

[39] Kavaliunas A, Stawiarz L, Hedbom J, Glaser A, Hillert J. The influence of immunomodulatory treatment on the clinical course of multiple sclerosis. Adv Exp Med Biol. 2015;822:19–24. 10.1007/978-3-319-08927-0_5 25416973

[40] Shirani A, Zhao Y, Karim ME, Evans C, Kingwell E, van der Kop ML, et al Association between use of interferon beta and progression of disability in patients with relapsing-remitting multiple sclerosis. JAMA. 2012;308(3):247–56. 10.1001/jama.2012.7625 22797642

