# Supplementary material for: Associations of Lifestyle, Medication, and Socio-Demographic Factors with Disability in People with Multiple Sclerosis: An International Cross-Sectional Study
Source: PLoS One. 2016 Aug 25;11(8):e0161701. doi: 10.1371/journal.pone.0161701 (PMC4999178; doi:10.1371/journal.pone.0161701)
Supplement: S1 Table — Relative risk ratios (RRR) and 95% Confidence Intervals (CI) obtained using multivariable logistic regression on multiply imputed data, model adjusted for age, gender and years since diagnosis. Statistically significant associations at a significance level of 0.05 are shown in bold. (DOCX) [file pone.0161701.s001.docx]

**S1 Table.**

|  | **Moderate disability** | |  | **Major disability** | |  |
| --- | --- | --- | --- | --- | --- | --- |
|  | **RRR** | **95% CI** | **p-value** | **RRR** | **95% CI** | **p-value** |
| Latitude (degrees) | **1.02** | **(1.01,1.04)** | **<0.001** | 1.02 | (1,1.04) | 0.1 |
| BMI |  |  |  |  |  |  |
| Underweight | 1.13 | (0.65,1.91) | 0.7 | 0.87 | (0.37,2.04) | 0.75 |
| Overweight | 1.02 | (0.79,1.31) | 0.9 | 0.83 | (0.54,1.27) | 0.39 |
| Obese | 1.07 | (0.81,1.43) | 0.6 | 0.99 | (0.62,1.58) | 0.97 |
| Alcohol consumption |  |  |  |  |  |  |
| Moderate or high | 0.79 | (0.64,1) | 0.1 | **0.4** | **(0.27,0.58)** | **<0.001** |
| Comorbidities |  |  |  |  |  |  |
| None |  |  |  |  |  |  |
| One | 1.21 | (0.93,1.59) | 0.15 | 1.05 | (0.67,1.66) | 0.82 |
| Two | **1.45** | **(1.08,1.94)** | **0.01** | 1.05 | (0.64,1.71) | 0.84 |
| Three or more | **1.41** | **(1.04,1.89)** | **0.02** | 0.76 | (0.45,1.25) | 0.28 |
| DHQ (per 30 points) | **0.66** | **(0.49,0.88)** | **<0.01** | 0.6 | (0.4,1) | 0.1 |
| Smoker |  |  |  |  |  |  |
| No |  |  |  |  |  |  |
| Current or former | **1.31** | **(1.07,1.61)** | **<0.01** | 1.06 | (0.75,1.49) | 0.73 |
| Vitamin D supplementation |  |  |  |  |  |  |
| low |  |  |  |  |  |  |
| high | 1.03 | (0.79,1.35) | 0.83 | 1.42 | (0.91,2.21) | 0.12 |
| IPAQ |  |  |  |  |  |  |
| low |  |  |  |  |  |  |
| high | **0.36** | **(0.3,0.45)** | **<0.001** | **0.09** | **(0.06,0.13)** | **<0.001** |
| Omega3 supplementation |  |  |  |  |  |  |
| none |  |  |  |  |  |  |
| flaxseed only | 0.95 | (0.6,1.4) | 0.81 | **0.43** | **(0.21,0.89)** | **0.02** |
| other | 0.9 | (0.7,1.1) | 0.26 | 0.72 | (0.5,1.05) | 0.1 |
| DMD use |  |  |  |  |  |  |
| Not taken >12 months | |  |  |  |  |  |
| Taken >12 months | 0.85 | (0.7,1.04) | 0.13 | **0.52** | **(0.34,0.77)** | **0.001** |
