# Supplementary material for: Associations of Lifestyle, Medication, and Socio-Demographic Factors with Disability in People with Multiple Sclerosis: An International Cross-Sectional Study
Source: PLoS One. 2016 Aug 25;11(8):e0161701. doi: 10.1371/journal.pone.0161701 (PMC4999178; doi:10.1371/journal.pone.0161701)
Supplement: S2 Table — Odds ratios (OR) and 95% Confidence Intervals (CI) obtained using multivariable logistic regression on multiply imputed data, model adjusted for age, gender, and years since diagnosis. Statistically significant associations at a significance level of 0.05 are shown in bold. (DOCX) [file pone.0161701.s002.docx]

**S2 Table.**

| Factor | OR | 95% CI | p-value |
| --- | --- | --- | --- |
| Disability |  |  |  |
| Mild | Reference |  |  |
| Moderate | **1.88** | **(1.42,2.49)** | **<0.001** |
| Major | **2.18** | **(1.08,4.4)** | **0.03** |
| Latitude (degrees) | 1 | (0.99,1.02) | 0.62 |
| BMI |  |  |  |
| Underweight | 0.64 | (0.35,1.17) | 0.15 |
| Overweight | 1.17 | (0.88,1.57) | 0.23 |
| Obese | 1.15 | (0.84,1.58) | 0.38 |
| Alcohol consumption |  |  |  |
| Moderate or high | 0.97 | (0.76,1.23) | 0.79 |
| Comorbidities |  |  |  |
| None |  |  |  |
| One | 1.32 | (0.98,1.77) | 0.1 |
| Two | **1.6** | **(1.15,2.22)** | **0.01** |
| Three or more | **2.36** | **(1.68,3.32)** | **<0.001** |
| DHQ (per 30 points) | 0.72 | (0.51,1.01) | 0.06 |
| Smoker |  |  |  |
| No |  |  |  |
| Current or former | 0.93 | (0.73,1.11) | 0.54 |
| Vitamin D supplementation |  |  |  |
| low |  |  |  |
| high | 0.82 | (0.61,1.23) | 0.2 |
| IPAQ |  |  |  |
| low |  |  |  |
| high | 0.95 | (0.73,1.05) | 0.71 |
| Omega3 supplementation |  |  |  |
| none |  |  |  |
| flaxseed only | 0.65 | (0.4,1.67) | 0.1 |
| other | 1.28 | (0.97,1.86) | 0.1 |
| DMD use |  |  |  |
| Not taken for more than 12 months | |  |  |
| Taken for more than 12 months | **0.49** | **(0.38,0.6)** | **<0.001** |
